# Natural infection of SARS-CoV-2 variant XBB.1.9.1.4.1 in laboratory Syrian hamsters

**DOI:** 10.1128/spectrum.01862-24

**Published:** 2025-02-11

**Authors:** Chunmao Zhang, Zhendong Guo

**Affiliations:** 1 Changchun Veterinary Research Institute, Chinese Academy of Agricultural Sciences, Changchun, China; National Microbiology Laboratory, Winnipeg, Manitoba, Canada

**Keywords:** SARS-CoV-2, XBB, natural infection, laboratory animals, Syrian hamster

## LETTER

At least 18 different animal species, spanning pets, farm animals, captives, and wildlife, have been reported to be infected with SARS-CoV-2 (1). These outbreaks have raised concerns about the potential for human-to-animal, animal-to-animal, and animal-to-human transmission. Notably, the Syrian hamster serves both as a household pet and as an experimental animal model. As a good small animal model, Syrian hamster has been used to study the pathogenesis and transmissibility of SARS-CoV-2 (2, 3), as well as to evaluate the efficacy of vaccines and antiviral drugs (4, 5). Syrian hamsters can also be experimentally infected by different SARS-CoV-2 variants, such as Alpha, Beta, Delta, and Omicron variants (6–12). Recently, two research teams from Hongkong University independently reported the potential transmission of the SARS-CoV-2 Delta strain from pet hamsters to humans (13, 14). Despite these findings, there have been no documented cases of natural SARS-CoV-2 infection in laboratory Syrian hamsters. Here, we presented the first report of natural infection with SARS-CoV-2 Omicron variant XBB.1.9.1.4.1 in laboratory Syrian hamsters.

**Fig 1:**
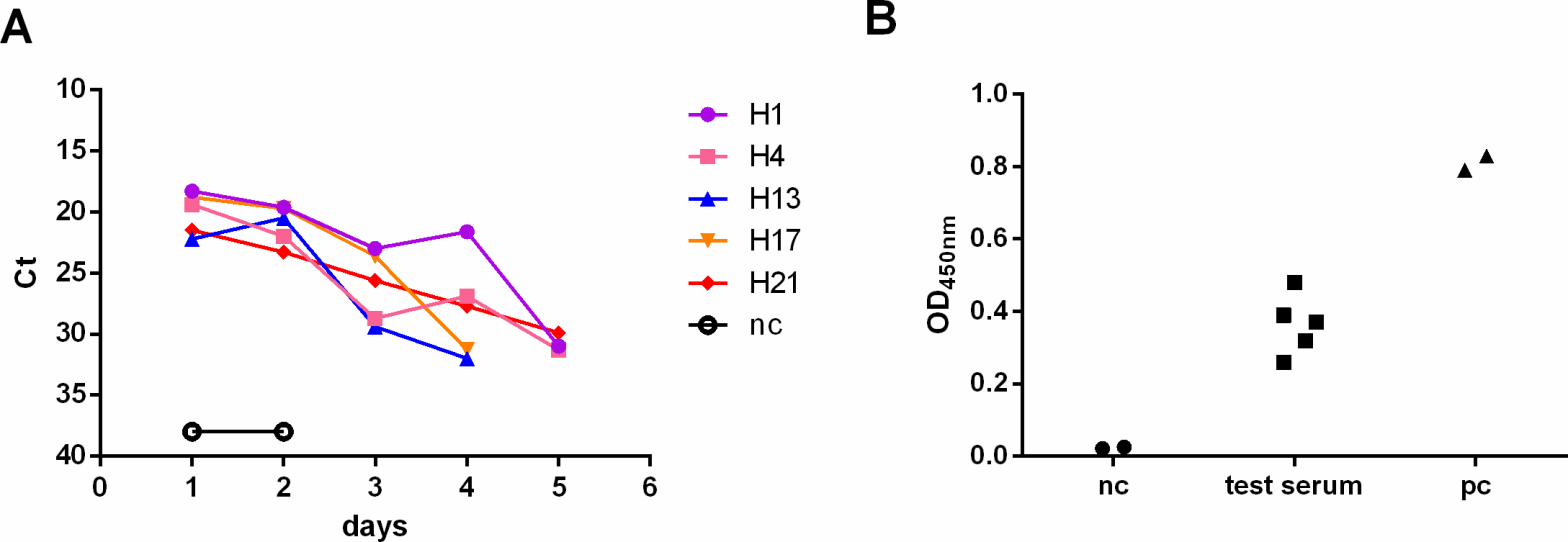
The cycle threshold values (Ct) for nasal washes and OD_450nm_ for N protein antibody detection in hamster serum. (A) nc represents negative control of naive hamster nasal washes. (B) nc represents negative control of two naive hamster serum, and pc represents positive control.

In September 2023, prior to commencing our animal experimental study, we collected nasal wash samples from a batch of newly ordered Syrian hamsters and promptly conducted routine SARS-CoV-2 screening using a rapid antigen test kit. Unexpectedly, five of these samples tested strongly positive for SARS-CoV-2. To further assess the viral presence, we sequentially collected nasal washes from these five hamsters and determined their viral load using RT-qPCR. Nasal washes of two naive hamsters from another batch ordered before were set as the negative control. In the first 2 days, Ct values in nasal wash samples were below 22, indicating a relatively high viral load, which subsequently gradually reduced to a significantly lower level (Fig. 1A).

Fourteen days after the initial positive tests, serum samples were collected from these five Syrian hamsters, and all serum samples tested positive for SARS-CoV-2 N protein antibody by ELISA (Fig. 1B). Moreover, for nasal wash samples with Ct values less than 22, we conducted virus isolation using Vero-E6 cells, and live SARS-CoV-2 was isolated from nasal washes of hamster H1. The full viral genome was then amplified by multiple overlapping PCR reactions using a panel of primers specific for SARS-CoV-2 and sequenced using an ABI3730XL sequencer. Using the Nextstrain platform, we identified the pangolin lineage of the isolated virus, which belonged to the FL.4.11 clade. The unaliased name assigned to this particular variant was XBB.1.9.1.4.1.

In summary, we have reported the first natural infection of the SARS-CoV-2 Omicron variant XBB.1.9.1.4.1 in laboratory Syrian hamsters, as well as the successful isolation and characterization of this virus. Given the highly experimental susceptibility of Syrian hamsters to a series of SARS-CoV-2 variants (Alpha, Beta, Gamma, Delta, and Omicron) and the emerging variants (6–12, 15), and considering the transmission of Delta variant AY.127 from pet hamsters to humans, which led to a pet-shop-related COVID-19 outbreak in Hong Kong (13, 14), it is imperative to strengthen the monitoring of COVID-19 in laboratory animals, pet animals, and wild animals and reinforce biosafety management measures for laboratory animals.

## SUPPLEMENTAL MATERIAL

Supplemental material spectrum.01862-24-s0001.docx Supplemental methods and Table S1.

ASM does not own the copyrights to Supplemental Material that may be linked to, or accessed through, an article. The authors have granted ASM a non-exclusive, world-wide license to publish the Supplemental Material files. Please contact the corresponding author directly for reuse.
