# Supplementary material for: Natural infection of SARS-CoV-2 variant XBB.1.9.1.4.1 in laboratory Syrian hamsters
Source: Microbiol Spectr. 2025 Feb 11;13(3):e01862-24. doi: 10.1128/spectrum.01862-24 (PMC11878043; doi:10.1128/spectrum.01862-24)
Supplement: Supplemental material — Supplemental methods and Table S1. [file spectrum.01862-24-s0001.docx]

**Title:** Natural Infection of SARS-CoV-2 Variant XBB.1.9.1.4.1 in Laboratory Syrian Hamsters

**Authors and affiliations:**

Chunmao Zhang^1^*, Zhendong Guo^1^

1Changchun Veterinary Research Institute, Chinese Academy of Agricultural Sciences, Changchun, China

.**^*^Correspondence author:**

Chunmao Zhang: [jk704715@sina.com](mailto:jk704715@sina.com)

Tel:0431-66869875

Mailing address: Yujinxiang Street, No 573, Changchun, China, 130122

**Supplementary Materials**

**Materials and Methods**

**Sampling and Screening for SARS-CoV-2**

Nasal washes were collected from newly ordered 6-week-old male hamsters using 1 mL of phosphate-buffered saline (PBS). The SARS-CoV-2 N protein rapid antigen testing kits (YHLO BIOTECH, Shenzhen) were used for SARS-CoV-2 screening. The tests were performed according to manufactures’ instruction for use with slight modification. Briefly, 100 uL of the supernatants from the collected samples were gently mixed with 100 uL of the antigen extraction buffer and incubated for 2 minutes. This mixture was then added into the sample pad of the rapid antigen test strip. After a waiting period of 15 minutes, the test results were carefully read.

**Cell culture, virus isolation and CPE assay**

Vero-E6 cells (CRL1586, ATCC, USA) were cultured in high-glucose Dulbecco’s modified Eagle’s medium (DMEM; Sigma Aldrich, USA), supplemented with 10% fetal bovine serum (FBS; Sigma Aldrich, USA), 100 U / mL penicillin and100 μg/ml streptomycin, at 37 ℃ with 5% CO2. As Vero-E6 cells were grown as a monolayer in 6-well plates and reached 80% confluence, the culture medium was discarded, and 1 mL of the nasal washes of hamsters was added to each well. After one hour’s incubation at 37℃, the nasal washes were aspirated, and 3 mL of DMEM supplemented with 2% fetal bovine serum. was added to each well. The cytopathic effect (CPE) was monitored daily under a microscopy for three consecutive days. We mainly focused on the status of cell rounding and the relative number of rounding cells. The culture supernatant was subsequently used for SARS-CoV-2 antigen detection, RNA extraction and viral genome sequencing.

**Genome sequencing**

The viral RNA was reverse transcribed into cDNA with the FastKing RT Kit KR116 (Qiagen, Germany), and this cDNA was subject to multiple overlapping PCR reactions for full genome amplification with a panel of primers specific for SARS-CoV-2. The primer pairs are listed in Table S1.The PCR was performed with2×PhantaMax Master Mix(P525, Vazyme). The PCR reaction conditions comprised an initially predenatured at 95℃ for 3 min, and then 35 cycles of 95℃ for 15 s, 55℃ for 15 s, 72℃ for 1min, and finally 72℃for 10 min. The PCR amplicons were then sequenced using the ABI3730XL sequencer and assembled into a complete viral genome. The sequence of the viral genome was deposited to GISAID, with the identified number of EPI_ISL_19388134.

**Viral RNA quantification**

100 uL of the nasal washes was used to extract viral RNA using the Simply P total RNA Extraction Kits (BioFlux, Hangzhou). The extracted viral RNA was eluted with 50 uL of the RNase-free water, and subsequently 5 uL of the RNA elute was used for real-time qPCR to detect the N gene of SARS-CoV-2. The primers sequences for the N gene were: forward primer-GGGGAACTTCTCCTGCTAGAAT, reverse primer-CAGACATTTTGCTCTCAAGCT, and the probe-5'-FAM-TTGCTGCTGCTTGACAGATT-TAMRA-3. The experiments were performed with an ABI7500 system (Roche,Switerland) using One Step PrimeScript^TM^ III RT–qPCR Mix (catalogue No: RR600A, Takara). The amplification reaction conditions comprised an initial reverse transcriptase step at 50℃ for 20 min, followed by 95℃ for 3 min, and then 45 cycles of 95℃ for 5 s, 57℃ for 45 s, and finally 25℃ for 10 min.

**SARS-CoV-2 N protein antibody detection by Enzyme-linked immunosorbent assay**

The Hamster anti-SARS-CoV-2 N protein IgG ElISA kit (FineTest, Wuhan) was utilized for antibody detection in hamster serum. The test was performed following the manufactures’ instruction. Briefly, 50 uL of diluted hamster serum samples were added to wells precoated with SARS2 N protein and incubated at 37℃for 30 minutes. After two washes with 350 uL of washing buffer, 50 uL of biotin labeled antibody dilutions were added to the wells and incubated again at 37 ℃for 30 min. Following three washes, 50 uL of HRP-streptavidin conjugate dilutions were added to the wells and incubated for another 30 min at 37℃. After five washes, 50 uL of 3,3',5,5'-tetramethylbenzidine (TMB) substrate was added for coloring and incubated for 10-20 minutes. Finally, a stop solution was added to terminate the enzymatic reaction, and the optical density (OD) values were read at 450 nm.The serum samples of two naive hamsters were as the negative control (nc), and the SARS-CoV-2 N protein antibody (100 ng/mL) in the kit was as the positive control (pc).

Table S1 the PCR primer pairs for viral genome amplification and sequencing

| Primer | Sequence |
| --- | --- |
| hCoV-19-F1 | TTGTAGATCTGTTCTCTAAACGAAC |
| hCoV-19-R1 | GCCAGATTCATTATGGTATTCG |
| hCoV-19-F2 | ACCAAATGTGCCTTTCAACTC |
| hCoV-19-R2 | AGCAACATAAGCCCGTTAATAC |
| hCoV-19-F3 | GAAACATTTGTCACGCACTCA |
| hCoV-19-R3 | AGGAATCTCAGCGATCTTTTG |
| hCoV-19-F4 | ACGAAGTTCTACTTGCACCATTA |
| hCoV-19-R4 | CCATTAACTTGTGGGTATTTC |
| hCoV-19-F5 | AGAGAAGTGAGGACTATTAAGGTGTT |
| hCoV-19-R5 | CAGGCAAGATTATCCATTCCC |
| hCoV-19-F6 | CCCTGACTTAAATGGTGATGTG |
| hCoV-19-R6 | ACTATCAACGATGTAAGAAGACTGG |
| hCoV-19-F7 | TGCATGTTGTAGACGGTTGT |
| hCoV-19-R7 | AAGTTTTGATGGTGTGTAACAGATG |
| hCoV-19-F8 | AGGATACAAGGCTATTGATGGTG |
| hCoV-19-R8 | CCAAAGACCGTTAAGTGTAGTTGT |
| hCoV-19-F9 | ATTTTAGTGGAGCAATGGATACAAC |
| hCoV-19-R9 | ACGAGTGTCAAGACATTCATAAGTG |
| hCoV-19-F10 | AATATGGTCTATATGCCTGCTAGTTG |
| hCoV-19-R10 | AACCTACCTCCCTTTGTTGTG |
| hCoV-19-F11 | ATTGTGGGAAATCCAACAGGT |
| hCoV-19-R11 | CGTTCACCTAAGTTGGCGTAT |
| hCoV-19-F12 | CACGTCAACGTCTTACTAAATACAC |
| hCoV-19-R12 | CCCAACCCATAAGGTGAGG |
| hCoV-19-F13 | ACGTAATGTCATCCCTACTATAACTC |
| hCoV-19-R13 | AGTCAGTAACATTATCGCTACCAAC |
| hCoV-19-F14 | AATTAGTCTTGTCTGTTAATCCGTA |
| hCoV-19-R14 | CTTTTCTCCAAGCAGGGTTAC |
| hCoV-19-F15 | ACGCACATTGCTAACTAAGGG |
| hCoV-19-R15 | ACTCGTGGACAGCTAGACACC |
| hCoV-19-F16 | AGCGCACCTGTTGTCTATGTG |
| hCoV-19-R16 | ACACCATTACGGGCATTTCTA |
| hCoV-19-F17 | AATACTCAATAATTTGGGTGTGGAC |
| hCoV-19-R17 | AGTAACAAAGGCTGTCCACCA |
| hCoV-19-F18 | CTCATTATTAGTGATATGTACGACCC |
| hCoV-19-R18 | CAATAGATTCTGTTGGTTGGAC |
| hCoV-19-F19 | CTTGCTTTACATAGAAGTTATTTGAC |
| hCoV-19-R19 | TGTGGGTATGGCAATAGAGTTA |
| hCoV-19-F20 | CTTACTCCTACTTGGCGTGTTTA |
| hCoV-19-R20 | TGACAAATGGCAGGAGCAGT |
| hCoV-19-F21 | AAATCAGAGCTTCTGCTAATCTTG |
| hCoV-19-R21 | GTTCTTCAGGCTCATCAACAAT |
| hCoV-19-F22 | CAGGTGATGGCACAACAAGTC |
| hCoV-19-R22 | AGTAACCTGAAAGTCAACGAGATG |
| hCoV-19-F23 | AAGGACCTGCCTAAAGAAATC |
| hCoV-19-R23 | TTCATTTTACCGTCACCACC |
| hCoV-19-F24 | GCACTCCGCATTACGTTTGG |
| hCoV-19-R24 | GGTGGCTCTTTCAAGTCCTC |
